# Intelligent Emergency Stop Algorithm for a Manipulator Using a New Regression Method

**DOI:** 10.3390/s120607451

**Published:** 2012-05-31

**Authors:** Minkyu Cheon, Jeisung Lee, Wonju Lee, Chang-Ho Hyun, Mignon Park

**Affiliations:** 1 The School of Electrical and Electronic Engineering, Yonsei University, 134 Shinchon-Dong, Seodaemun-Gu, Seoul 120-749, Korea; E-Mails: 1000minkyu@yonsei.ac.kr (M.C.); leejaisung@yonsei.ac.kr (J.L.); delicado@yonsei.ac.kr (W.L.); 2 The School of Electrical Electronic and Control Engineering, Kongju National University, 275 Budae-Dong, Seobuk-Gu, Cheonan, Chungnam 331-717, Korea; E-Mail: hyunch@kongju.ac.kr

**Keywords:** emergency stop algorithm, risk and inefficiency, regression method

## Abstract

In working environments with large manipulators, accidental collisions can cause severe personal injuries and can seriously damage manipulators, necessitating the development of an emergency stop algorithm to prevent such occurrences. In this paper, we propose an emergency stop system for the efficient and safe operation of a manipulator by applying an intelligent emergency stop algorithm. Our proposed intelligent algorithm considers the direction of motion of the manipulator. In addition, using a new regression method, the algorithm includes a decision step that determines whether a detected object is a collision-causing obstacle or a part of the manipulator. We apply our emergency stop system to a two-link manipulator and assess the performance of our intelligent emergency stop algorithm as compared with other models.

## 1. Introduction

Increasing the safety of robots, especially industrial manipulators, is just as important as improving their performance. A collision between a manipulator and a person, for example, may cause severe personal injury as well as damage to the machinery. Thus, it is necessary to develop an algorithm that can detect collisions before they occur and make the manipulator stop before damage is done.

Various emergency stop or obstacle avoidance algorithms for robots, particularly those utilizing distance-measuring sensors [1–4] or vision sensors have been reported [5–8] and those algorithms using each type of sensor have advantages and disadvantages. For example, a vision sensor provides information such as colors (RGB), edges, and other various features that are useful for image processing; however, it does not provide a distance value, which is instrumental for programming emergency stop capabilities. Conversely, a distance-measuring sensor determines the distance value between the sensor and another object, but it is limited in that it only measures the distance value. Consequently, if we use the distance measuring sensors for an emergency stop system, an intelligent algorithm is necessary that can predict a collision with obstacles. In this paper, we propose such a system.

In order to develop an efficient emergency stop algorithm, we consider two special cases in which the emergency stop is not needed. The first is the case that the detected object is not in the manipulator's path. The second is the case that detected object is not a collision-causing obstacle, *i.e.*, it is the part of the manipulator itself. Considering those two cases, our proposed algorithm prevents unnecessary stops and increases the efficiency of the manipulator.

An efficient emergency stop algorithm using a fuzzy lookup table method to determine whether a detected object is an obstacle was previously presented and showed good performance [9]. In this paper, however, we propose an emergency stop algorithm employing a new regression method that exhibits faster responses than the fuzzy lookup table method. This regression method is also more suitable for use in an emergency stop algorithm than other typical regression methods such as ridge regression and support vector regression. To implement our algorithm, we generate a function model of motor positions and measured distances using the new regression method. From this function model of sample data pairs, the distance value corresponding to a specific motor position can be predicted. The algorithm can then be used to determine whether the manipulator should stop by comparing the real distance value (as measured by the sensor) to the estimated distance value (as calculated by the function model). We further introduce our system in Section 2, demonstrate a new regression method in Section 3, and assess the experimental performance of our proposed system in Section 4.

## 2. Efficient Emergency Stop System

In this section, we propose an intelligent emergency stop system for manipulators using distance-measuring sensors.

### 2.1. Efficient Emergency Stop Algorithm

**Figure 1.**
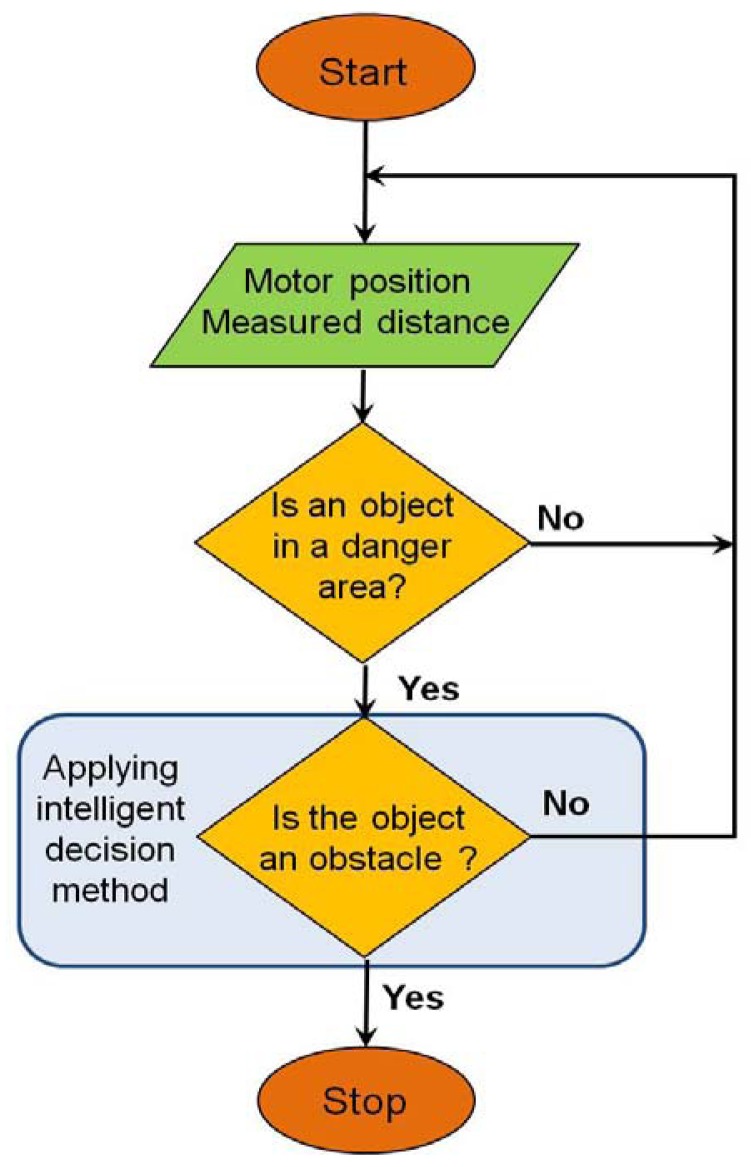
Flowchart of the proposed efficient emergency stop algorithm. After an object is detected in the danger area, the algorithm determines whether the object is an obstacle or a part of the manipulator.

Figure 1 shows the flowchart of our proposed efficient emergency stop algorithm. First, the proposed algorithm ascertains whether an object is located in a danger area via the distance values measured by the sensors. This danger area is established by a predetermined danger distance, and can be set as a predefined fixed value, or it can be a variable area that depends on the location of the sensor or operating velocity of the manipulator.

If one or more objects are sensed in the danger area, the algorithm verifies whether the detected object is a part of the manipulator (e.g., a link) or an obstacle that could cause a collision with the machine. If the detected object is judged to be an obstacle, the manipulator is shut down.

### 2.2. Consideration of the Direction of Movement

In this section, we consider the direction of movement of the manipulator in order to develop an efficient emergency stop algorithm. Obviously, accidents can occur if there is an obstacle—e.g., a person—in the manipulator's path; however, the manipulator does not need to stop when it does not move towards the obstacle.

Hence, our emergency stop algorithm, which considers the direction of motion of the manipulator, not only solves the safety problem but also helps to establish a more efficient operation of the manipulator.

**Figure 2.**
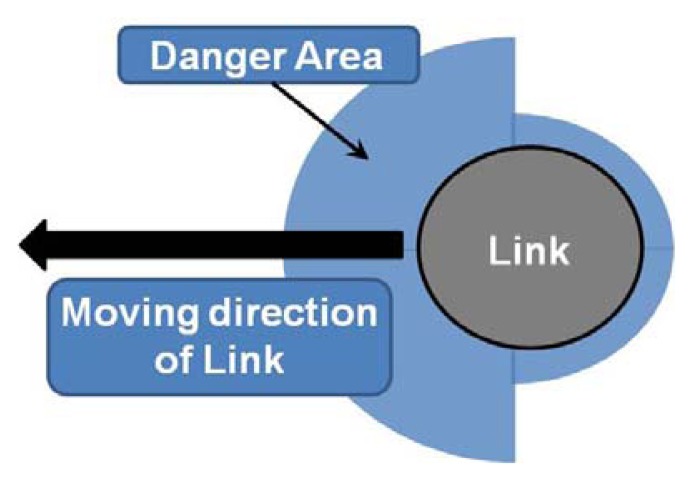
Consideration of moving direction.

Figure 2 shows the danger area of a link, which is generated only in the moving direction of the manipulator. In other words, only the distance values measured by the sensors located on the side of the manipulator that is in direction of motion are transferred to the system, whereas distance values from sensors on the other side of the manipulator (opposite the direction of motion) are ignored [9].

### 2.3. Intelligent Decision Method

The information provided by the distance-measuring sensor, e.g., an ultrasonic and an infrared sensor, is limited to a distance value, which is not sufficient information for the emergency stop algorithm to distinguish a part of the manipulator from true obstacles. Consequently, this limitation not only affects the efficient operation of the manipulator since it cannot work at specific motor positions where sensors detect parts of the manipulator in danger areas, but it also can result in unnecessary stops.

In order to solve this problem, we propose an intelligent decision method that can determine whether the sensed object is an obstacle that could cause a collision with the manipulator or not. We develop the intelligent decision method by applying a new regression method, which we introduce in Section 3.

## 3. Sum of Risk and Inefficiency Minimization

For the decision step mentioned in Section 2.3, we apply a new regression method to the emergency stop algorithm. The regression method generates a function model of the motor positions and distance values, and then the function is used to predict a distance value. Next, the algorithm compares this predicted (estimated) distance value to a real distance value measured by a sensor and determines whether a stop is necessary. In this section, we introduce our proposed regression method—*i.e.*, the sum of risk and inefficiency (SRI) minimization.

### 3.1. Regression Method

The regression method determines the relationship between variables, and then uses that information to predict unknown variables. More specifically, the regression method generates an approximated function model using sample data (variables), and values of specific variables can be estimated by the function model. We call this function model the regression model, and the linear regression model has the form:

(1) $$y\approx f(\text{w},\text{x})={\text{w}}^{t}\text{x}$$

where **x** is an input vector (an independent variable), *y* is a real-valued output (a dependent variable), and **w** is parameters of the regression model. Various regression methods, e.g., ridge regression, and support vector regression have been widely used in machine learning fields including robotics [10–12].

In order to apply a regression method to our emergency stop algorithm, we need a training procedure that will generate a function model of the motor position and distance values—hence the use of sample data pairs for this purpose. We collect motor position and corresponding distance data pairs by operating the manipulator under working conditions, but without obstacles. Because we cannot collect every motor position and distance data pair, we collect data at regular intervals of the position. We then apply the motor position values as inputs and the distance values as outputs to a regression method and a function model is generated for each sensor. After a function model for each sensor is generated by the regression method, distance values corresponding to specific motor position values can be estimated. Note that the distance-measuring sensors used for our experiment are ultrasonic sensors, which are typical distance-measuring sensors and have been widely employed in robotic applications [13,14].

### 3.2. Permissible Error Range

**Figure 3.**
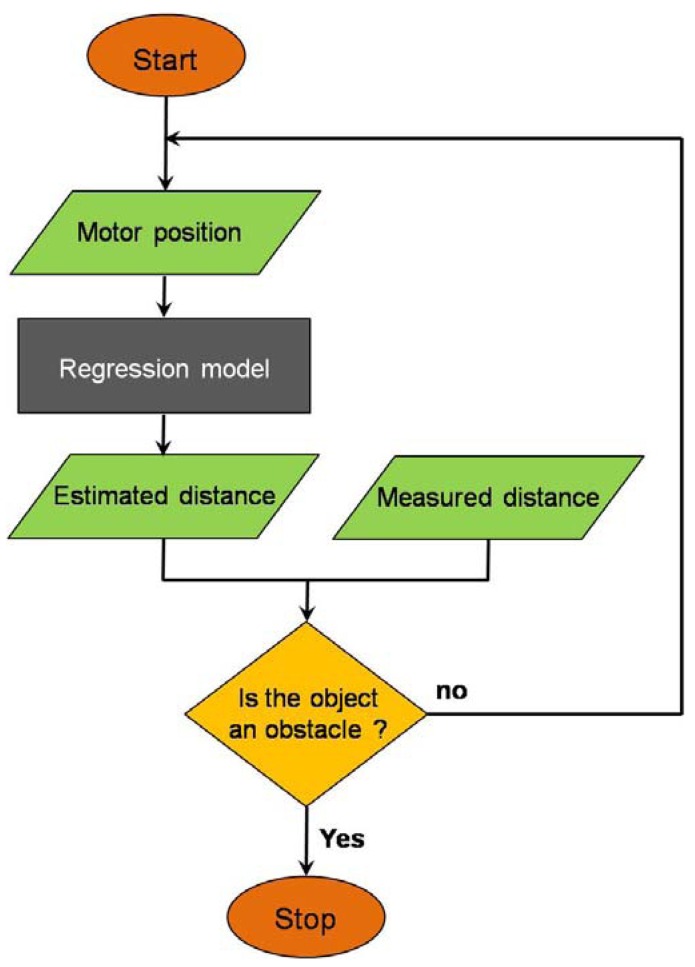
Intelligent decision step: The algorithm determines whether to stop or not by comparing a distance value estimated by the regression model to a distance value measured by the sensor.

After an object is sensed in a danger area, the algorithm determines whether the object is an obstacle or not by using the new regression method, as shown in the flowchart in Figure 3. As discussed, a function model for each sensor is generated by the regression method using data pairs (motor position and distance value) collected under working conditions without obstacles, which can be used to estimate a distance value for specific motor position. The algorithm regards the detected object as an obstacle when the distance value measured by the sensor is smaller than the estimated distance value.

**Figure 4.**
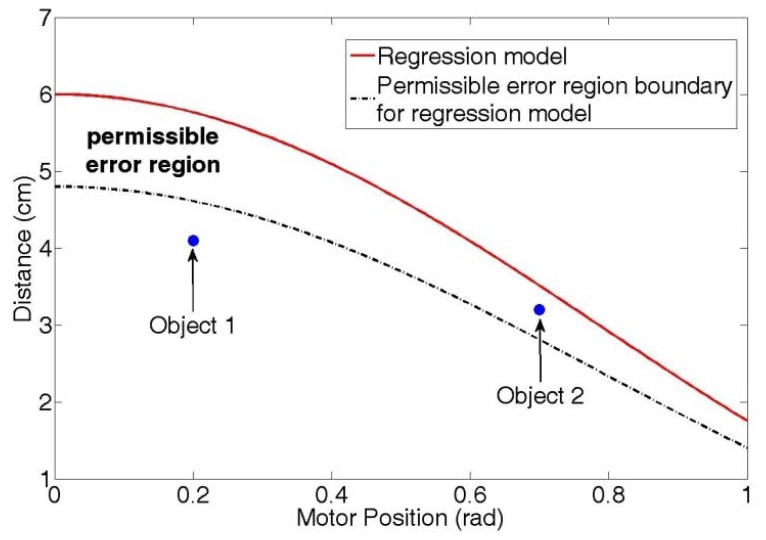
Permissible error region.

Because the function model generated by the regression method is approximated by sample data pairs, the estimated distance values provided by the regression model are not identical to the real distance values, producing experimental error. Consequently, when the difference between a measured value and an estimated value is larger than the allowed error range, the detected object is regarded as an obstacle that is not a part of the manipulator. We call this allowed error range the “permissible error region”, and note that it is not fixed region but is directly proportional to the distance values, as shown in Figure 4.

The black dotted line represents the permissible error region boundary of the regression model (red line), and region between the red line and the blue dotted line is the permissible error region. For object 1 in Figure 4, the manipulator stopped because the object was located outside the permissible error region. Conversely, for object 2 in Figure 4, the manipulator did not stop because the object was located inside the permissible error region. In this paper, we define the lower bound of the permissible error region as 90% of distance values. In other words, the permissible error region is 10% of the distance values. We determine the region by trial and error.

### 3.3. Analysis of Risk and Inefficiency

**Figure 5.**
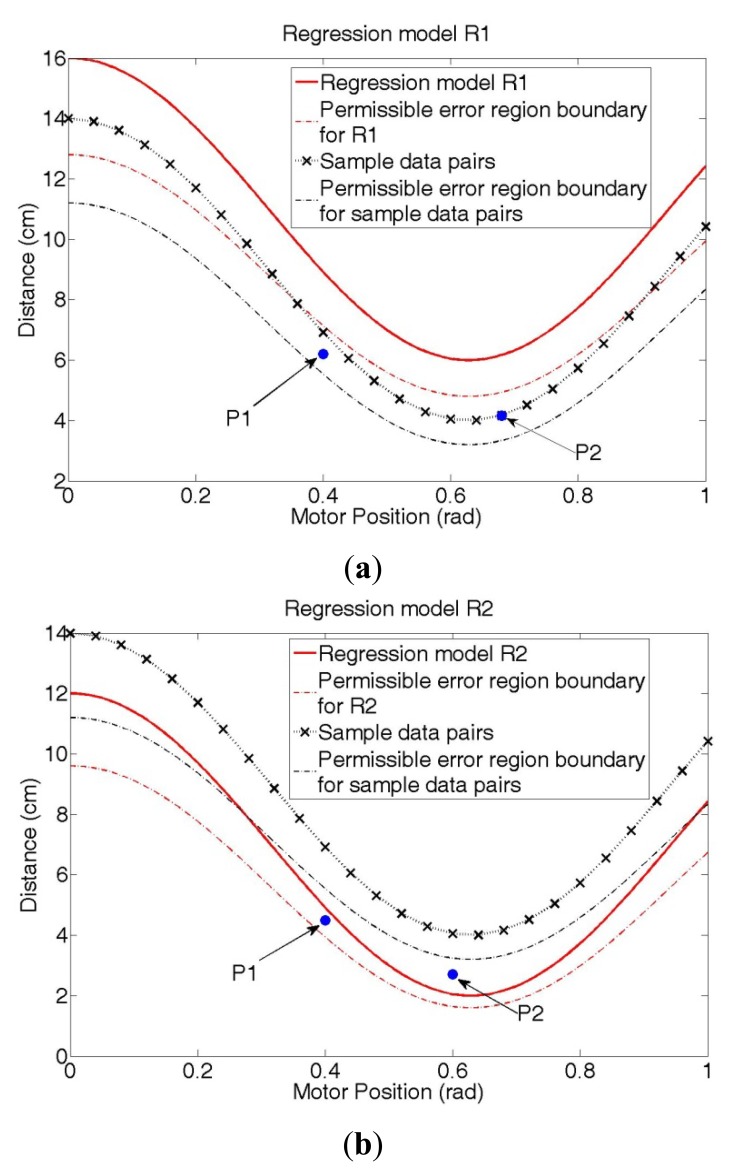
Two different regression results: (**a**) Regression model R1—Estimated distance values are larger than measured distance values. (**b**) Regression model R2—Estimated distance values are smaller than measured distance values.

In Figure 5(a,b), R1 and R2 are the results of two different regression methods. We assume that all the data pairs are located in danger areas and that the black x points are sample data pairs used to obtain the regression function model.

In Figure 5(a), P1 is located inside the permissible error range for sample data pairs, and P2 is included in the sample data pairs, which means that P2 is a part of the manipulator; therefore, the manipulator does not need to stop operating in the cases of P1 and P2. However, if the emergency stop algorithm is designed by the regression model R1, then the manipulator will stop working because both P1 and P2 are outside of the permissible error region for the model R1. In other words, the emergency stop algorithm associated with the R1 regression model decreases the efficiency of the operation because it results in unnecessary stops.

In Figure 5(b), P1 and P2 are detected outside the permissible error region for sample data pairs, so for these cases, the manipulator should stop. Yet, if the regression model R2 from Figure 4(b) is applied to the emergency stop algorithm, the manipulator will not stop because P1 is inside the permissible error range for R2, and the distance value of P2 is larger than the estimated distance value from R2 at the same motor position. Consequently, the emergency stop algorithm associated with the R2 regression model increases the risk of the operation.

In summary, if the value estimated by a regression model is larger than the real distance value, as shown in Figure 5(a), the inefficiency increases; on the contrary, if the estimated distance value is smaller than the real distance value, as shown in Figure 5(b), the risk increases.

### 3.4. Sum of Risk and Inefficiency Minimization

**Figure 6.**
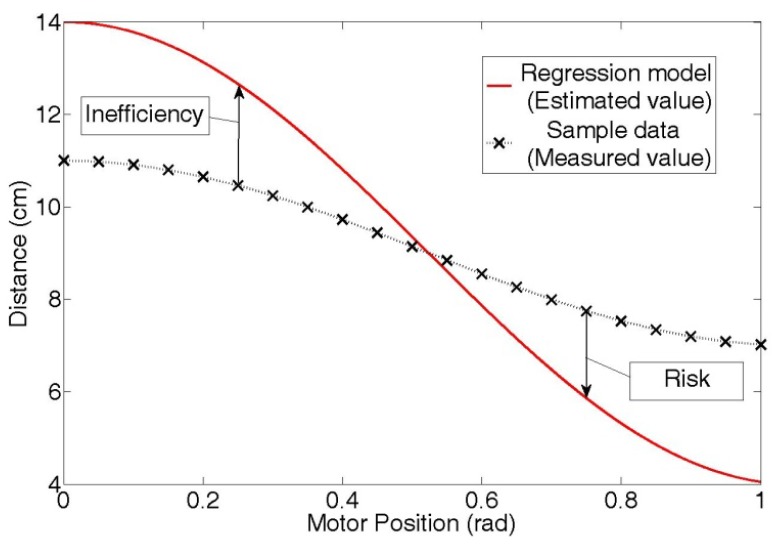
Risk and inefficiency.

In response to this paradox, we propose a new regression method in this section that minimizes the SRI. As shown in Figure 6, risk is defined as an error that is smaller than the real distance value. Likewise, inefficiency is an error that is larger than the real distance value. These risk and inefficiency values increase as the difference between a real distance and an estimated distance value of the regression model increases.

The risk and inefficiency can be represented as follows:

(2) $$\text{risk}=\{\begin{array}{cc} \alpha_{r}(t_{n}-{\text{w}}^{t}{\text{x}}_{n}) & ift_{n}>{\text{w}}^{t}{\text{x}}_{n}\\ 0 & \text{otherwise} \end{array}$$

(3) $$\text{inefficiency}=\{\begin{array}{cc} \alpha_{i}({\text{w}}^{t}{\text{x}}_{n}-t_{n}) & if{\text{w}}^{t}{\text{x}}_{n}>t_{n}\\ 0 & \text{otherwise} \end{array}$$

where *α_r_* and *α_i_* are proportional parameters for the risk and the inefficiency terms, respectively, **w** is a parameter of the regression model, **x***_n_* is a sample motor position, and *t_n_* is a sample distance value measured by a specific sensor corresponding to the position **x***_n_*. Thus, **w***^t^***x***_n_* is the estimated distance value from the regression model. The dimension of **x***_n_* depends on the number of motors, which affects the measured distance value *t_n_*. For example, **x***_n_* is at least a three-dimensional vector if the number of motors that affect the measured distance value *t_n_* is three. In addition, the dimension of **x***_n_* can be expanded if a basis expansion, e.g., a polynomial representation, is applied. Note that the dimension of **w** is the same as **x***_n_*. Our proposed regression method minimizes the SRI and can be represented as follows:

(4) $$\begin{array}{l} \underset{\text{w}}{\arg\min}\text{SRI}(\text{w},\text{x},t)=\underset{\text{w}}{\arg\min}\sum_{\text{all}n}\left[L_{\alpha_{r}}[t_{n}-{\text{w}}^{t}{\text{x}}_{n}]+L_{\alpha_{i}}[{\text{w}}^{t}{\text{x}}_{n}-t_{n}]\right] \end{array}$$

where the function *L* is a loss function that is zero when the input is less than zero and a linear function with a slope of *α* (*α_r_* and *α_i_*) when the input is larger than zero. The optimal solution of Equation (4), however, cannot be obtained because the function *L* is not differentiable. Therefore, we replace the function *L* with a quadratic function that can be differentiated.

**Figure 7.**
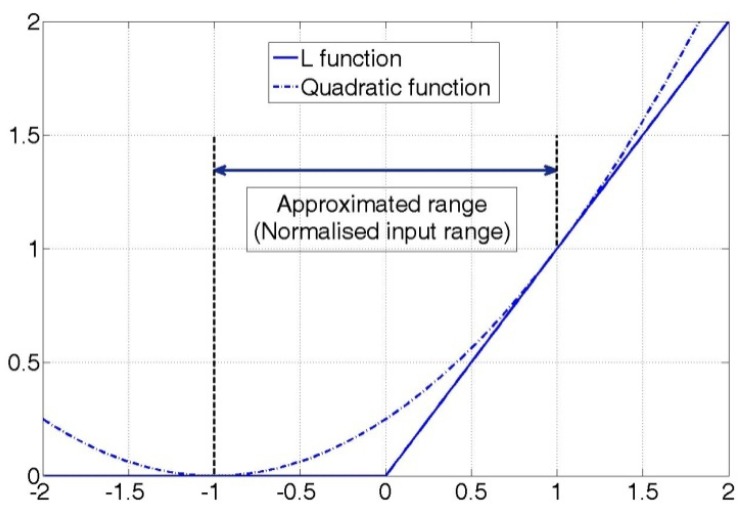
Loss function *L* and an approximated quadratic function.

In Figure 7, the solid line is the *L* function with a slope of 1, and the dotted line is a quadratic function. If all of the inputs are normalized to be within a specific range, the *L* function can be approximated by the quadratic function as shown in Figure 7. We can apply a similar quadratic function with the *L* function by adjusting its coefficients and moving it on the horizontal axis. The quadratic function in Figure 7 has been shifted from the origin by a value of −1 on the horizontal axis, and its second order coefficient is 0.5.

By substituting the quadratic function in Figure 7 for the linear *L* function (*Lα_r_* and *Lα_i_*) in Equation (4), the equation can be reformulated as follows:

(5) $$\begin{array}{l} \underset{\text{w}}{\arg\min}\text{SRI}(\text{w},\text{x},t)=\underset{\text{w}}{\arg\min}b{\left\|\text{w}\right\|}_{2}^{2}+\sum_{\text{all}n}\beta_{r}{\left\{(t_{n}-{\text{w}}^{t}{\text{x}}_{n})+\eta_{r}\right\}}^{2}+\sum_{\text{all}n}\beta_{i}{\left\{({\text{w}}^{t}{\text{x}}_{n}-t_{n})+\eta_{i}\right\}}^{2} \end{array}$$

where *β_r_* and *β_i_* are the second order coefficients, and *η_r_* and *η_i_* are the moving distances on the horizontal axis of the quadratic function for the risk and the inefficiency terms, respectively. *b*‖**w**‖^2^_2_ is the regularized term, and *b* is a small positive real number.

We also consider the fact that the risk and inefficiency values should adapt to the real measured distance value, *t_n_*, because the permitted error range is small when *t_n_* is small. In other words, the risk and inefficiency value should be relatively large when a distance value measured by a sensor is small and *vice versa*. Therefore, we add another parameter *s_n_* to Equation (5):

(6) $$\begin{array}{l} \underset{\text{w}}{\arg\min}\text{SRI}(\text{w},\text{x},t,s)=\underset{\text{w}}{\arg\min}b{\left\|\text{w}\right\|}_{2}^{2}+\sum_{\text{all}n}C_{1}s_{n}\beta_{r}{\left\{(t_{n}-{\text{w}}^{t}{\text{x}}_{n})+\eta_{r}\right\}}^{2}+\sum_{\text{all}n}C_{2}s_{n}\beta_{i}{\left\{({\text{w}}^{t}{\text{x}}_{n}-t_{n})+\eta_{i}\right\}}^{2} \end{array}$$

where *s_n_* is a value between 0 and 1, and is inversely proportional value to *t_n_*. *C_1_* and *C_2_* are weight values for the risk and inefficiency, respectively, and are always larger than 0. By controlling the values of *C_1_* and *C_2_*, we vary the weight of the risk or the inefficiency, and can obtain a proper optimal solution according to the working situations. If the algorithm is applied to extremely dangerous working conditions, the risk is a significantly greater factor than the inefficiency. In this condition, *C_1_* than *C_2_* should be given larger values so that we obtain a regression model result with a smaller risk. We present the regression results for various values of *C_1_* and *C_2_* in Section 4.1.

The optimality condition of Equation (6) is given by:

(7) $$\frac{\partial \text{SRI}(\text{w},\text{x},t,s)}{\partial \text{w}}=0$$

which implies that:

(8) $$b\text{w}+\sum_{\text{all}n}-C_{1}s_{n}\beta_{r}{\text{x}}_{n}\{(t_{n}-{\text{w}}^{t}{\text{x}}_{n})+\eta_{r}\}+\sum_{\text{all}n}C_{2}s_{n}\beta_{i}{\text{x}}_{n}\{({\text{w}}^{t}{\text{x}}_{n}-t_{n})+\eta_{i}\}=0$$

Then, Equation (8) can be rearranged as:

(9) $$\left[bI+C_{1}\beta_{r}\sum_{\text{all}n}{{\text{x}}_{n}}^{t}\cdot {\text{x}}_{n}\cdot s_{n}+C_{2}\beta_{i}\sum_{\text{all}n}{{\text{x}}_{n}}^{t}\cdot {\text{x}}_{n}\cdot s_{n}\right]\text{w}=\left[C_{1}\beta_{r}\sum_{\text{all}n}{\text{x}}_{n}\cdot t_{n}\cdot s_{n}+C_{2}\beta_{i}\sum_{\text{all}n}{\text{x}}_{n}\cdot t_{n}\cdot s_{n}\right]+\left[C_{1}\eta_{r}\beta_{r}\sum_{\text{all}n}{\text{x}}_{n}\cdot s_{n}-C_{2}\eta_{i}\beta_{i}\sum_{\text{all}n}{\text{x}}_{n}\cdot s_{n}\right]$$

Finally, the optimal solution of the regression parameter **w** can be written as follows:

(10) $$\begin{array}{l} \text{w}= \end{array}\begin{array}{l} {\left[bI+(C_{1}\beta_{r}+C_{2}\beta_{i})\sum_{\text{all}n}s_{n}{\text{x}}_{n}{{\text{x}}_{n}}^{t}\right]}^{-1}\times \left[(C_{1}\beta_{r}+C_{2}\beta_{i})\sum_{\text{all}n}t_{n}s_{n}{\text{x}}_{n}+(C_{1}\eta_{r}\beta_{r}-C_{2}\eta_{i}\beta_{i})\sum_{\text{all}n}s_{n}{\text{x}}_{n}\right] \end{array}$$

## 4. Experiments

**Figure 8.**
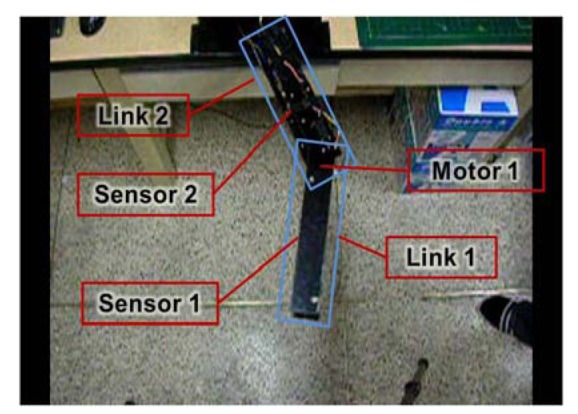
Two-link manipulator.

**Figure 9.**
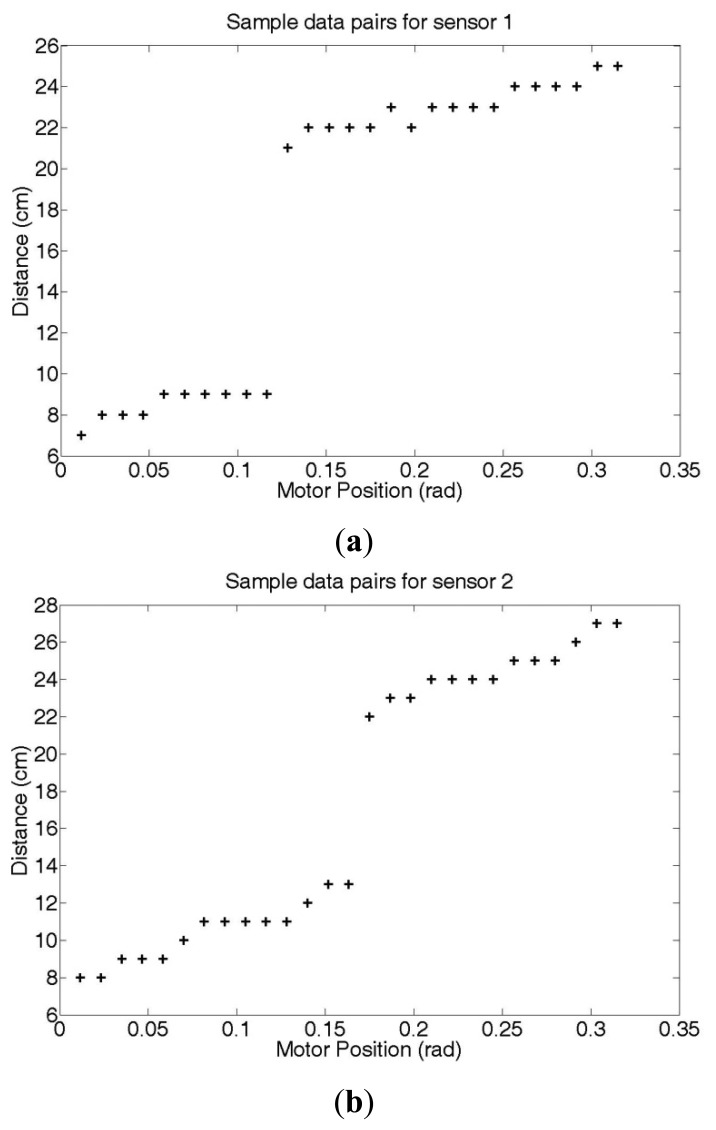
Sample motor position and measured distance data pairs for a training procedure of the new regression method for (**a**) sensor 1 (**b**) sensor 2.

For performance verification of our proposed emergency stop algorithm, we apply the algorithm to a two-link robot and attach two ultrasonic sensors to each link, as shown in Figure 8. We consider a specific situation where sensors 1 and 2 detect links 2 and 1, respectively, in danger areas. We collect data pairs that represent the position values of motor 1 and the distance values, as measured by sensors 1 and 2, under the condition without obstacles. From those data pairs, we extract pairs whose measured distance values are smaller than the danger area distance and apply those pairs to the regression method. (Note that we set the distance of the danger area to 30 cm.) Those data pairs are shown in Figure 9.

We assume that *x_i_* is the position value of motor 1 for the i^th^ data pair, and that *t_i_*_1_ and *t_i_*_2_ are the distance values measured by sensors 1 and 2 of the i^th^ data pair, respectively. We generate two regression function models of the data pairs (*x_i_*, *t_i_*_1_) and (*x_i_*, *t_i_*_2_).

As shown in Figure 9(a,b), there are drastic changes in the measured distance values. These changes occur due to the angular measuring range of the ultrasonic sensors. In other words, when a sensor strays out of its optimal angular measuring range to measure a specific part of the manipulator, the measured distance value rapidly increases as shown in Figure 9(a,b).

### 4.1. Regression Model

We construct regression models using SRI minimization to determine whether a detected object is an obstacle or not. Two regression models are generated—one for sensor 1 and another for sensor 2. We apply the motor position vectors **x***_i_* = (1, *x_i_*, *x_i_^2^*, *x_i_^3^*, …, *x_i_^p^*) which are applied by the polynomial basis expansion instead of the one-dimensional position value of the motor *x_i_*. This polynomial representation of the basis expansion can help obtain a more complex and detailed regression result compared to that of a model applying the one-dimensional input.

**Figure 10.**
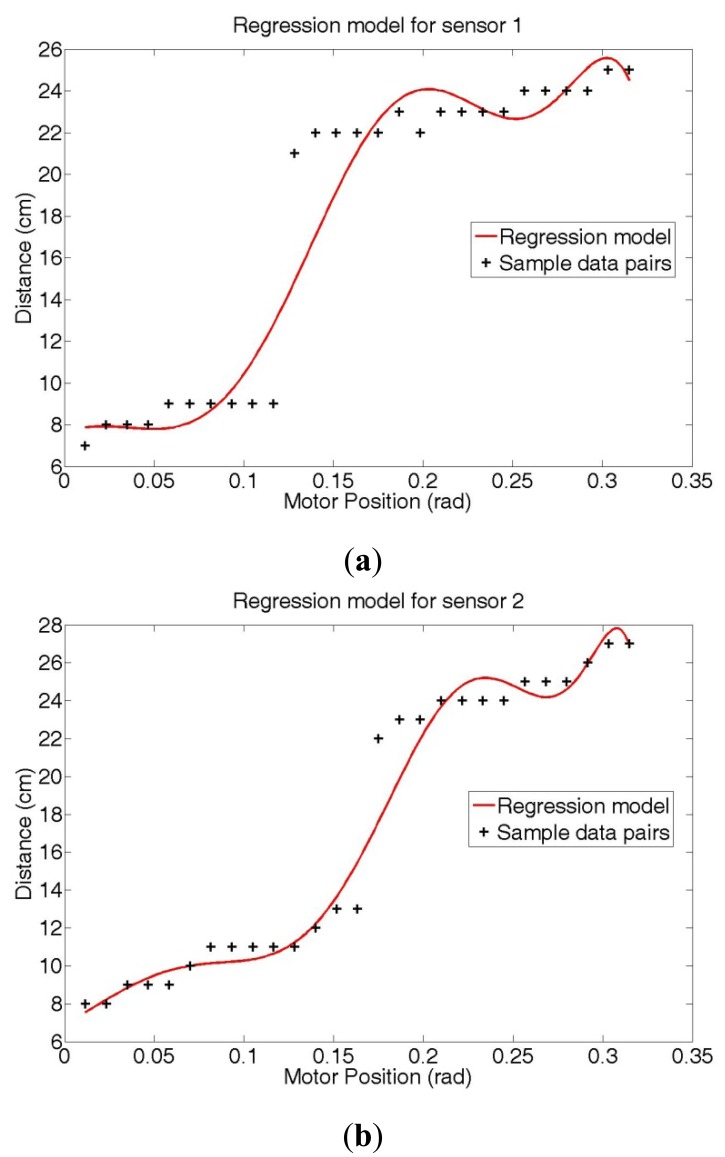
Function models generated by the SRI regression method for (**a**) sensor 1 (**b**) sensor 2 (*p* = 10).

Figure 10(a,b) shows the SRI regression results using 11-dimensional polynomial motor position vectors (*p* = 10) [note that we set *β_r_* = *β_i_* = 0.5, *η_r_* = *η_i_* = 1, and *C_1_* = *C_2_* = 1 in Equation (10)].

**Figure 11.**
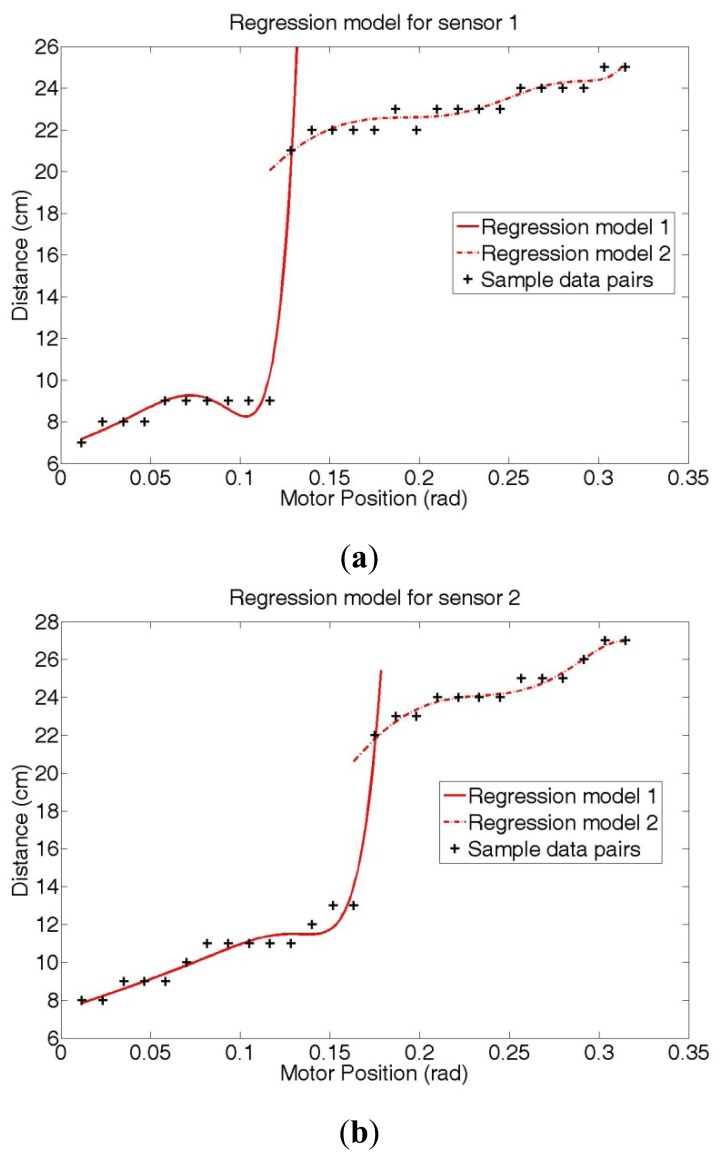
Function models generated by two SRI regression methods for (**a**) sensor 1 (**b**) sensor 2 (*p* = 10).

As shown in Figure 10(a,b), the error between the measured distance and the estimated distance values is relatively large near the section where measured distance values rapidly change. This error is a consequence of the curve fitting method using a linear model, which is a difficult method to obtain a step-like function model. Therefore, we divide the sample data pairs into two sections and obtain two regression models: Figure 11(a,b) shows the results obtained by two regression models that applied the proposed SRI minimization for sensors 1 and 2, respectively.

Finally, we compare the results of the proposed SRI regression, ridge regression, support vector regression, and fuzzy lookup table methods [9] using 1,000 test data pairs (test data samples are collected in the same way as training samples). Table 1 shows the results of each method. Note that the proposed SRI regression using two models shows the best performance. (*I.e.*, it shows the smallest root-mean-squared error and root-mean-squared error multiplied by *s_n_*).

For implementation as an emergency stop algorithm, the decision speed is one of the most important factors. Hence, the decision time of the system using the fuzzy lookup table method applying five membership functions is 874.38 μs, and is 1,074.32 μs when the lookup table method with 10 membership functions is applied; however, when we apply regression methods, e.g., ridge regression, support vector regression and SRI regression, the decision time averages only 6.37 μs.

Table 2 shows the SRI regression result according to different parameters *C_1_* and *C_2_* in Equation (10). It also shows that risk and inefficiency results can be controlled by adjusting the *C_1_* and *C_2_* values. In other words, relatively large *C_1_* values can decrease the risk whereas large *C_2_* values can decrease the inefficiency.

### 4.2. Experiment Using Two-Link Manipulator

**Figure 12.**
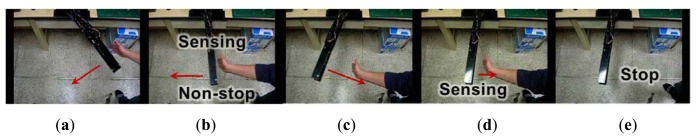
(**a–e**) By considering the direction of motion; (b) the manipulator does not stop when it does not move towards an obstacle; however, (d,e) the manipulator stops when an obstacle is in the manipulator's path.

We apply our proposed emergency stop algorithm to a two-link manipulator with ultrasonic sensors (Figure 8) and verify the performance of the algorithm. Figure 12(a–e) shows that the algorithm considers the direction of motion of the manipulator. For example, when the manipulator does not move towards an obstacle as shown in Figure 12(b), it keeps moving; yet, the manipulator stops when it moves towards an obstacle, as shown in Figure 12(d,e).

**Figure 13.**
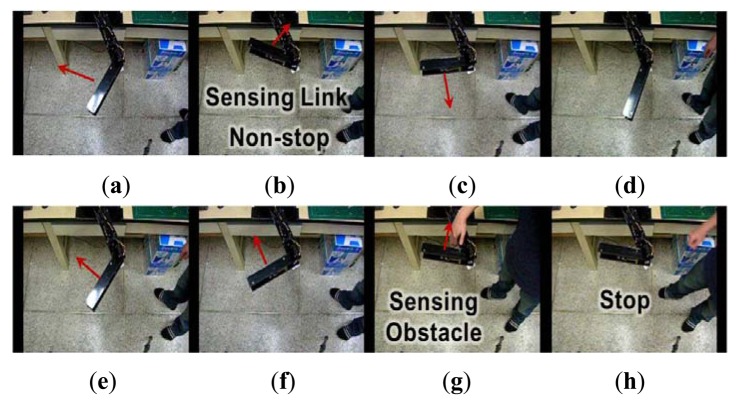
(**a–h**) Using an intelligent decision step, (b) the manipulator keeps moving if the sensor detects a part of the manipulator (e.g., a link) in the danger area, but (g,h) the manipulator stops when an obstacle is detected in the danger area.

Figure 13(a–h) shows that the algorithm recognizes whether a sensed object is an obstacle or a part of the manipulator. In Figure 13(b), an object is sensed, but the manipulator keeps operating because the algorithm classifies it as a part of the manipulator. On the other hand, when another obstacle is sensed, as shown in Figure 13(g), the manipulator stops operating since the algorithm correctly recognizes that the detected object could cause a collision.

## 5. Conclusions

In this paper, we have proposed an emergency stop system for manipulators using distance-measuring sensors. Our proposed intelligent emergency stop algorithm considered the direction of motion of the manipulator. In addition, it was able to decide whether the detected objects were obstacles that could cause a collision with the manipulator or not by applying a new regression method. We applied our system to a two-link manipulator and verified the performance of our algorithm by comparing it with other regression methods. Consequently, we expect that the application of our emergency stop algorithm will enhance the safety and efficiency of working environments with industrial robots.

## Figures and Tables

**Table 1.** Regression results of fuzzy lookup table method, ridge regression, support vector regression, and SRI regression (*p*=10, *β_r_* = *β_i_* = 0.5, *η_r_* = *η_i_* = 1, *C_1_* = *C_2_* = 1) using 1,000 test data. (unit: cm).

**Method**		**RMS Error**		**RMS Error × *s****_n_*	

		**sensor 1**	**sensor 2**	**sensor 1**	**sensor 2**
Fuzzy lookup table	5 Fuzzy membership functions	0.9146	0.8403	0.5978	0.4423
	10 Fuzzy membership functions	0.5977	0.5219	0.2297	0.3395
Ridge regression	1 regression	1.1713	0.9134	0.7044	0.4606
	2 regression	0.3579	0.3595	0.2143	0.2355
Support vector regression	1 regression	1.4196	1.2219	0.66	0.6032
	2 regression	0.7497	0.677	0.3923	0.3595
SRI regression	1 regression	1.2804	0.8616	0.5922	0.3821
	2 regression	**0.3532**	**0.3546**	**0.2014**	**0.2304**

**Table 2.** SRI regression results according to different *C_1_* and *C_2_* values using 1,000 test data sets (*p*=10, *β_r_* = *β_i_* = 0.5, *η_r_* = *η_i_* = 1).

**Result**	**Sensor**	**SRI2 Regression Model**		

		**(*C_1_, C_2_*) = (1, 1)**	**(*C_1_, C_2_*) = (5, 1)**	**(*C_1_, C_2_)* = (1, 5)**
RMS error	1	**0.3532**	0.6744	0.7572
	2	**0.3546**	0.7767	0.7007
RMS risk	1	0.1752	**0.006**	0.7143
	2	0.129	**0.0063**	0.6352
RMS inefficiency	1	0.1781	0.6684	**0.0428**
	2	0.2257	0.7704	**0.0655**
RMS error × *s_n_*	1	**0.2014**	0.3626	0.3923
	2	**0.2304**	0.489	0.4286
RMS risk × *s_n_*	1	0.0921	**0.0049**	0.3607
	2	0.0804	**0.0047**	0.3838
RMS inefficiency × *s_n_*	1	0.1093	0.3577	**0.0317**
	2	0.15	0.4843	**0.0448**
